# A conserved role for the ESCRT membrane budding complex in LINE retrotransposition

**DOI:** 10.1371/journal.pgen.1006837

**Published:** 2017-06-06

**Authors:** Axel V. Horn, Ivana Celic, Chun Dong, Irena Martirosyan, Jeffrey S. Han

**Affiliations:** 1 Department of Biochemistry and Molecular Biology, Tulane University School of Medicine, New Orleans, LA, United States of America; 2 Department of Embryology, Carnegie Institution for Science, Baltimore, MD, United States of America; Fred Hutchinson Cancer Research Center, UNITED STATES

## Abstract

Long interspersed nuclear element-1s (LINE-1s, or L1s) are an active family of retrotransposable elements that continue to mutate mammalian genomes. Despite the large contribution of L1 to mammalian genome evolution, we do not know where active L1 particles (particles in the process of retrotransposition) are located in the cell, or how they move towards the nucleus, the site of L1 reverse transcription. Using a yeast model of LINE retrotransposition, we identified ESCRT (endosomal sorting complex required for transport) as a critical complex for LINE retrotransposition, and verified that this interaction is conserved for human L1. ESCRT interacts with L1 via a late domain motif, and this interaction facilitates L1 replication. Loss of the L1/ESCRT interaction does not impair RNP formation or enzymatic activity, but leads to loss of retrotransposition and reduced L1 endonuclease activity in the nucleus. This study highlights the importance of the ESCRT complex in the L1 life cycle and suggests an unusual mode for L1 RNP trafficking.

## Author summary

Long interspersed nuclear elements (LINEs) are a class of retrotransposable elements that mutate mammalian genomes. LINEs have been highly successful in the human genome, multiplying to over 800,000 copies. The LINE-encoded replication machinery is also used by other retrotransposons, and in total, has been responsible for the generation of over 1/3 of human DNA sequence. To replicate, a LINE mRNA forms a ribonucleoprotein particle (RNP) with its proteins. This RNP eventually enters the nucleus to integrate a cDNA copy of itself into chromosomes. The events between RNP formation and successful integration are difficult to study and largely unknown. Here we show that the ESCRT complex plays a conserved role in LINE retrotransposition in both yeast and humans. ESCRT is a membrane budding complex involved in cellular trafficking and membrane budding/fusion. Our results imply that membranes play an integral part of LINE replication, and ESCRT may be required for RNP trafficking towards the nucleus.

## Introduction

Long Interspersed Nuclear Elements (LINEs) are an ancient class of non-long terminal repeat (non-LTR) retrotransposable elements widely dispersed among eukaryotes. These elements can be categorized into distinct clades based on homology of conserved domains [1]. The L1 clade is of particular interest because its namesake element, L1, is widespread throughout mammalian genomes. L1 has been enormously successful in populating the human genome, comprising at least 17% of human DNA [2]. In addition, other human retrotransposons such as Alu and SVA depend on the L1 machinery to replicate [3–5]. When the sequences of these L1 “parasites” are taken into account, greater than 30% of the human genome has been produced by the L1 retrotransposition machinery [2].

**Fig 1:**
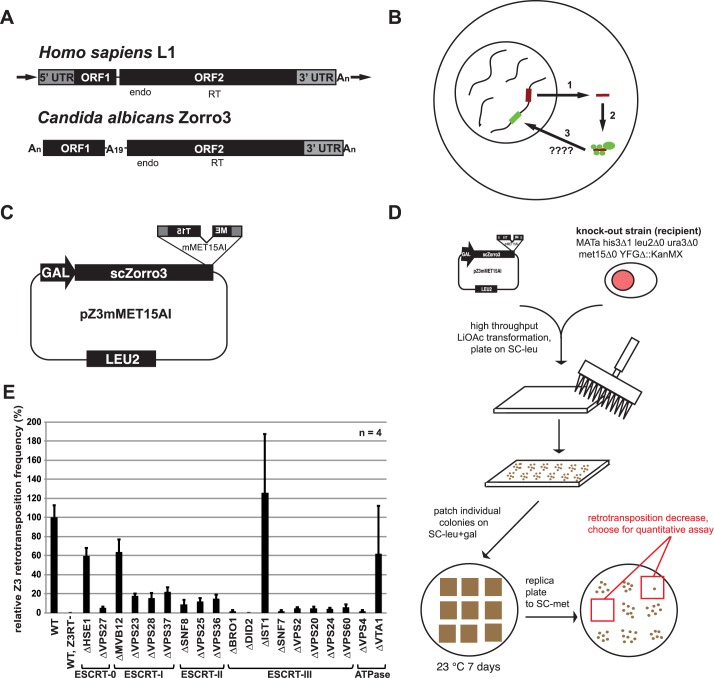
A screen for host factors affecting LINE retrotransposition. **A.** Comparison between the genomic structures of the two LINE elements used in this study. endo = endonuclease domain. RT = reverse transcriptase domain. arrows = target site duplications. **B.** Simple depiction of L1 replication cycle. 1) L1 mRNA is transcribed from a functional copy on the chromosomes. After export to the cytoplasm, 2) the mRNA is translated and the protein products associate with the mRNA to form an RNP. 3) the RNP enters the nucleus by an unknown mechanism, where a chromosome is nicked and reverse transcription occurs to generate a new insertion (green line). **C.** Zorro3 retrotransposition construct used for knockout collection screen. This assay is adapted from previously described assays [15,52,54,55]. **D.** Schematic to identify strains defective for Zorro3 retrotransposition. The retrotransposition construct was individually transformed into 4819 yeast strains, each knocked out for a different non-essential gene. Three transformants for each strain were assayed for retrotransposition as depicted, and those that appeared impaired in Met^+^ colony formation were selected to test using a quantitative retrotransposition assay [52]. **E.** ESCRT is required for efficient Zorro3 retrotransposition. Results of quantitative retrotransposition assay in strains deleted in various ESCRT genes. Results are normalized to the BY4741 (wt) strain. Error bars indicate standard deviations.

The structure of a typical active, full-length L1 is shown in Fig 1A. L1 has two open reading frames (ORFs) which encode two proteins called ORF1p and ORF2p. ORF1p is a homotrimeric non-sequence specific RNA binding protein that is thought to play a structural role in the L1 ribonucleoprotein particle (RNP) formation [6–11]. ORF1p also has nucleic acid chaperone activity *in vitro* [12]. The amino acids required for the chaperone activity are required for L1 retrotransposition [13], but how they contribute to L1 replication remains unclear. ORF2p encodes endonuclease and reverse transcriptase activity, both of which are important for retrotransposition [14–16].

To replicate, the bicistronic L1 mRNA is transcribed, translated, and complexes with L1-encoded proteins (ORF1p and ORF2p) to form a cytoplasmic L1 RNP [17,18] (Fig 1B). A cytoplasmic L1 RNP is presumed to gain access to the nucleus by an unknown mechanism. Once in the nucleus, ORF2p-encoded endonuclease activity nicks a chromosome, and ORF2p reverse transcriptase uses this nick as a primer to copy L1 mRNA at the new chromosomal site [19,20]. Together, these enzymatic events are referred to as target-primed reverse transcription (TPRT). How the TPRT complex is resolved has yet to be established. This entire process of L1 replication (transcription through integration) is called L1 retrotransposition or “L1 activity”. Sequencing efforts have shown that up to 30% of genomic structural variation between human individuals is due to L1 retrotransposition [21,22], and L1 retrotransposition is also a source of DNA damage [23,24] and novel disease alleles [25,26]. Thus, at a minimum, L1s are endogenous mutagens that have played a major role in the evolution of our genome. Recent studies have shown that the relationship between L1s and their mammalian host cells is even more complex, and that L1s may play a major unrecognized role in health and development. L1 overexpression is associated with infertility, cancer, neurological disorders, and aging [24,27–39], although whether L1 plays a biologically significant role in these processes is inconclusive.

The streamlined simplicity of an L1 element belies the complexity of LINE retrotransposition, suggesting that LINE RNPs utilize host factors to assist in their replication. Multiple proteomic studies and candidate based approaches have identified host factors that interact with and restrict L1 activity, such as the piwi-interacting RNA machinery [24,27,28], the APOBEC3 family [40–45], hnRNPL [46], and the antiviral factors MOV10 and ZAP [47–50]. However, the list of positive host factors (factors that facilitate L1 retrotransposition) with demonstrated specific and direct interaction with the L1 proteins is remarkably short. Currently we are aware of only one such positive factor that binds directly to L1 proteins. PCNA binds to ORF2p and is hypothesized to be recruited in the nucleus during or after L1 cDNA synthesis [49]. Another positive factor, the poly(A) binding protein PABPC1, binds the L1 RNP via RNA [51].

To facilitate the identification of positive LINE host factors, we previously developed a budding yeast model of LINE retrotransposition [52]. In this model, Zorro3, an L1-clade member from *Candida albicans* (Fig 1A), was redesigned for the ability to retrotranspose in *Saccharomyces cerevisiae*. We found that Zorro3 retrotransposes in a manner mechanistically similar to mammalian L1, and therefore we hypothesized that the *S*. *cerevisiae* genome encodes host factors required for LINE retrotransposition. In the current study, we describe a genetic screen using the Zorro3 model to identify host factors for LINE retrotransposition. We report the identification of the endosomal sorting complex required for transport (ESCRT) as a critical factor for successful LINE retrotransposition. ESCRT physically interacts with Zorro3, and the importance of ESCRT for retrotransposition is retained with human L1. Human ORF1p contains a conserved ESCRT-interacting domain which is dispensable for RNP formation and enzymatic function, but important for retrotransposition. Disruption of the ESCRT-L1 interaction alleviated L1 endonuclease-mediated toxicity. Together, our data reveals a surprising potential role for membrane budding in the trafficking of L1 RNPs.

## Results

### A screen for LINE retrotransposition host factors

To identify host factors important for LINE retrotransposition, we screened the *S*. *cerevisiae* yeast knockout collection [53] for strains unable to support robust Zorro3 retrotransposition. Each strain was transformed with a Zorro3 retrotransposition reporter plasmid (Fig 1C), then assayed for retrotransposition. This assay is based on previously described retrotransposition assays [52,54,55] and depends on the splicing of an artificial intron out of a reporter gene (mMET15AI) placed in the Zorro3 3’ untranslated region. A functional MET15 reporter is reconstituted only after Zorro3 transcription, splicing, and integration. Knockout strains were first qualitatively screened for lack of retrotransposition, then the identified retrotransposition-defective strains were quantitatively assayed for retrotransposition frequency (Fig 1D). Out of 4,819 knockout strains screened, 56 strains had a severe (> = 90%) decrease in retrotransposition as compared to a wild-type control strain (S1 Table). Of these 56 strains, 9 were deleted in a gene encoding an endosome associated protein, and 6 of these proteins belong to the endosomal sorting complex required for transport (ESCRT), a 27-fold enrichment of ESCRT genes as compared to the entire genome. Because our screen only identified 6 out of the 19 ESCRT proteins, we next individually tested deletion strains of all ESCRT genes with the quantitative retrotransposition assay, and found that virtually all ESCRT strains had a defect in retrotransposition (Fig 1E, S2 Table). The two exceptions, IST1Δ and VTA1Δ, have been reported as non-essential for ESCRT function [56,57]. In the early stages of our screen, we also transformed a plasmid expressing the lacZ gene under the control of the GAL1 promoter into selected knockout strains with potentially altered retrotransposition frequency. We assayed β-galactosidase activity in these strains to determine whether the altered retrotransposition frequencies could be explained by effects on the GAL1 promoter (S3 Table). For some knockout strains (e.g. SNO1, NPR1, YML095C-A, CUE1, YNL190W) reduction of β-galactosidase activity tracked closely with retrotransposition activity. In the case of the four ESCRT knockouts examined, β-galactosidase activity was lower, but could not entirely explain the >90% reduction in retrotransposition activity in each case (S3 Table). In sum, our screen suggests that ESCRT is required for efficient LINE retrotransposition in budding yeast.

### ESCRT physically interacts with Zorro3

**Fig 2:**
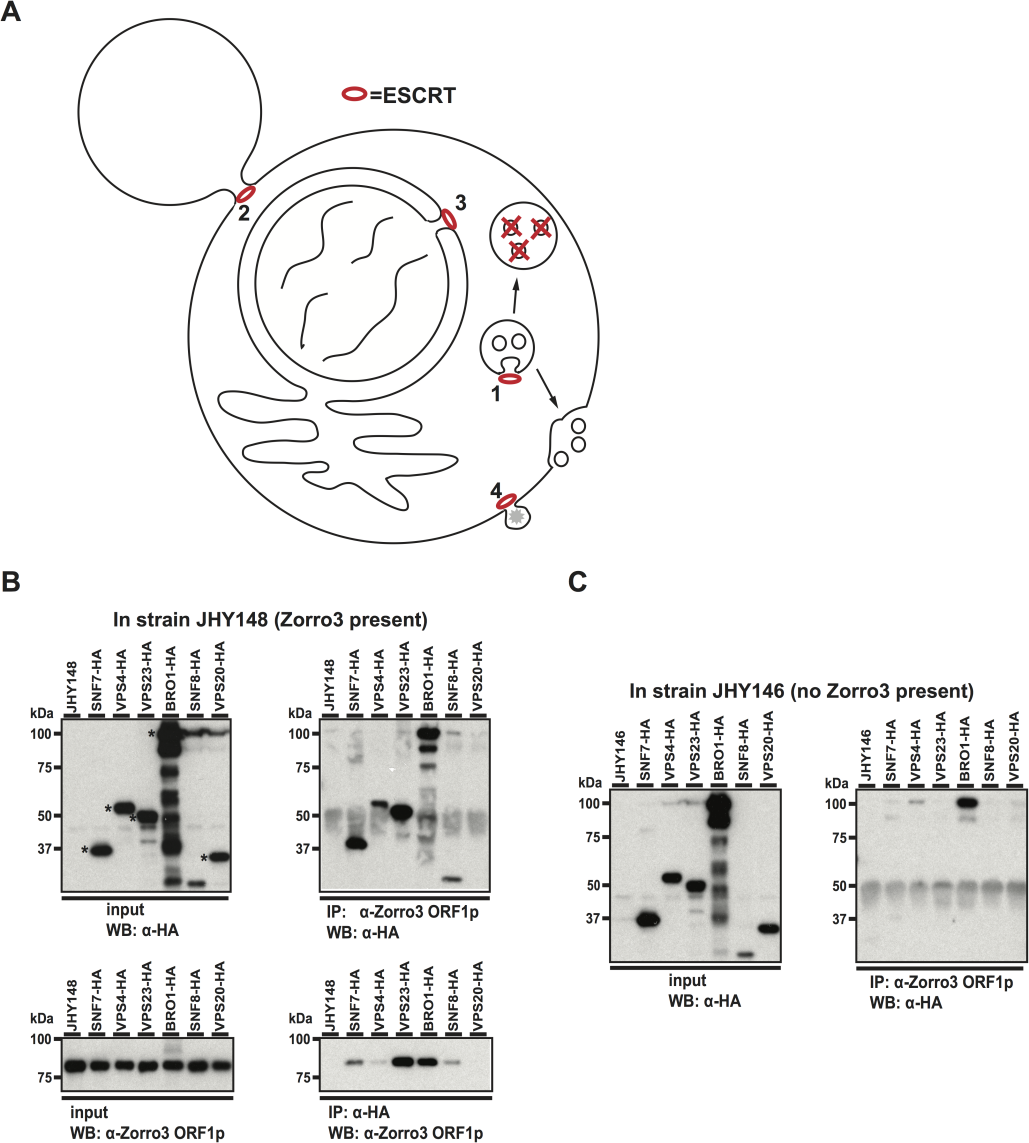
Interaction between ESCRT proteins and Zorro3 ORF1p. **A.** Known roles of ESCRT in the cell. 1) Formation of multivesicular bodies. 2) Cell abscission. 3) Nuclear membrane reformation after mitosis. 4) Viral budding. **B.** Immunoprecipitation in Zorro3 containing strains. JHY148 was transformed with plasmids encoding the indicated HA-tagged cDNAs. After galactose induction to express Zorro3 and the transformed cDNAs, cells were lysed and immunoprecipitations performed with anti-HA matrix or anti-Zorro3 ORF1p antibody. Whole lysates (left panels) or immunoprecipitations (right panels) were separated by SDS-PAGE and subjected to western blot with the indicated antibodies. Expected molecular weights of HA-tagged proteins are indicated with asterisks. **C.** Immunoprecipitation in control strains. JHY146 was transformed and analyzed as described in Fig 2B.

ESCRT has roles in cell abscission, formation of intraluminal vesicles, nuclear envelope reformation, and viral budding from the plasma membrane [58–65]. The commonality between these topologically equivalent processes is membrane fusion, a process ESCRT facilitates by constricting and sealing the membrane “neck” made in each case (Fig 2A). Because LINE RNPs have not been known to traffic through membranes, and ESCRT has multiple important cellular roles, we wondered whether ESCRT and Zorro3 directly interact, or if the retrotransposition defects present in ESCRT deficient strains were simply indirect genetic effects. To test this, we asked whether ESCRT proteins physically associate with Zorro3 proteins.

We HA-epitope tagged and overexpressed 6 yeast ESCRT proteins (Snf7p, Vps4p, Vps23p, Bro1p, Snf8p, and Vps20p) in the *S*. *cerevisiae* strain JHY148 [52], which expresses Zorro3 (Fig 2B). Snf7p, Vps4p, Vps23p, Bro1p, and Snf8p associated with Zorro3 ORF1p in both ORF1p and HA immunoprecipitations (Fig 2B). These associations were not disrupted in the presence of RNase A, suggesting that the interactions were not RNA mediated (S1 Fig). In the strain without a plasmid (JHY148 alone) or with empty vector (pGAL-HA, S1 Fig), HA pulldowns did not co-immunoprecipiate Zorro3 ORF1p, demonstrating that the interactions were dependent on the presence of HA-tagged ESCRT proteins. When the same experiment was performed in JHY146, a Zorro3-free strain isogenic to JHY148, the Zorro3 ORF1p antibody only pulled down Bro1p (Fig 2C), suggesting that while the Bro1p interaction is non-specific, the other ESCRT/ORF1p interactions are Zorro3 specific. Thus, components of the ESCRT complex physically interact with Zorro3.

### ESCRT proteins are required for efficient human L1 retrotransposition

**Fig 3:**
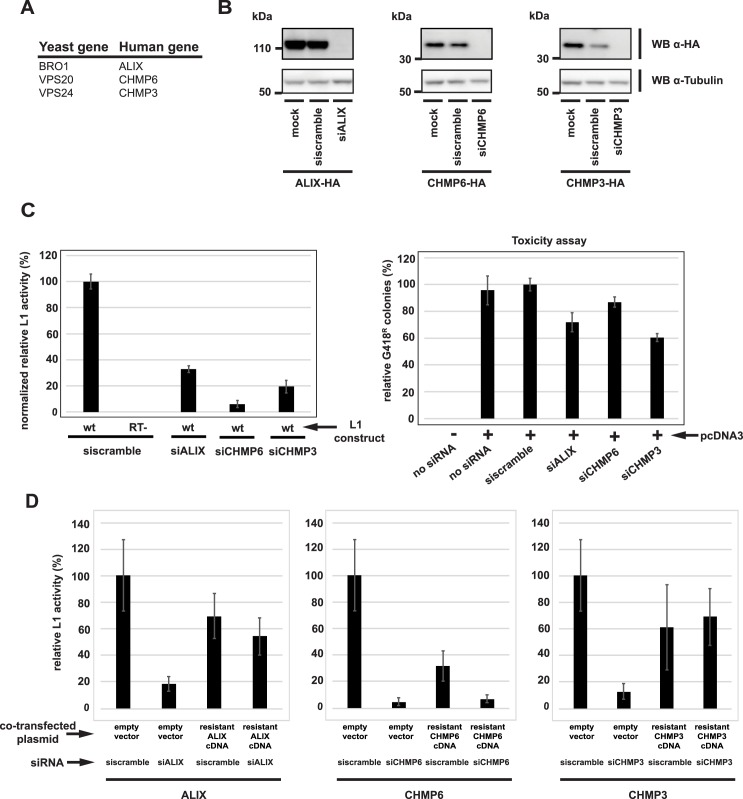
ESCRT proteins are required for efficient human L1 retrotransposition. **A.** Human genes (listed with the corresponding yeast ortholog) targeted for knockdown by siRNA in this figure. **B**. Validation of siRNA knockdown. HeLa cells were co-transfected with pEFALIX-2HA, pEFCHMP6-2HA, or pEFCHMP3-2HA and the indicated siRNAs. Shown are western blots of whole cell lysates. **C.** Knockdown of ESCRT genes reduces human L1 retrotransposition. HeLa cells were co-transfected with the indicated siRNA and L1 reporter plasmid, then selected with G418 for retrotransposition events. wt = pJM101L1.3. RT^-^ = pJM105L1.3. Results are relative to the wild type L1, and normalized to pcDNA3 induced colonies to take into account toxicity of ESCRT siRNAs. The ESCRT siRNA toxicity assay is shown in the right panel. Error bars indicate standard deviations. Raw data are shown in S4 Table. **D.** Rescue of ESCRT phenotypes. HeLa cells were co-transfected with pJM101L1.3 and the indicated plasmids and siRNAs, then selected with G418 for retrotransposition events. For each gene, results are normalized to pJM101L1.3 with scramble siRNA and pEF. Error bars indicate standard deviations. Raw data are shown in S5 Table.

**Fig 4:**
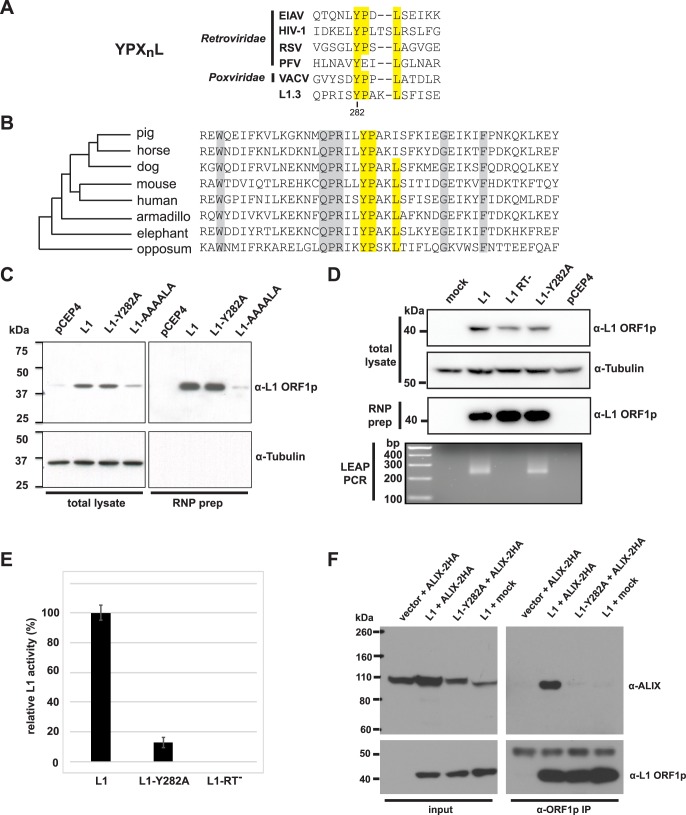
ESCRT/L1 interact via an L1 late domain. **A**. Comparison of L1 YPX_n_L motif to selected viral late domains. EIAV = equine infectious anemia virus. HIV-1 = human immunodeficiency virus-1. RSV = Rous sarcoma virus. PFV = prototype foamy virus. VACV = vaccinia virus. The Y282 residue of human L1 ORF1p is indicated. **B.** Conservation of YPX_n_L motif in mammals. Alignment of the YPX_n_L motif +/- 20 amino acids from indicated species, representing the breadth of mammalian diversity. Phylogenetic relationships are based on [104]. Consensus ORF1 sequences were derived from [105]. The YPX_n_L motif is highlighted yellow. Other conserved residues are highlighted gray. **C.** Expression levels and RNP purification of ORF1p mutants. Shown are western blots of whole cell lysates and RNP preparations from HeLa cells transfected with the indicated vectors. L1 = pJM101L1.3. L1-Y282A = pJM101L1.3-Y282A. L1-AAAALA = pJM110. **D.** Results of L1 element amplification protocol (LEAP) assays. Shown are western blots of whole cell lysates and RNP preps from transfected HeLa cells, and the resulting LEAP PCRs derived from the RNPs. **E**. Retrotransposition assays. HeLa cells were co-transfected with the indicated L1 reporter plasmids, then selected with G418 for retrotransposition events. L1 = pJM101L1.3. L1-Y282A = pJM101L1.3-Y282A. RT^-^ = pJM105L1.3. **F.** Co-immunoprecipitation of ORF1p and ALIX. tet-293T cells were transfected with pTetPuro (vector), pTetL1.3 (L1), or pTetL1.3–282 (L1-Y282A) and selected with puromycin. Puromycin selected cells were then transfected with pEFALIX-2HA. After ALIX transfection, cells were treated with doxycycline to induce L1 expression. 48 hours after ALIX transfection, cells were lysed and ORF1p immunoprecipitated with the antibody JH74. Shown are western blots of the lysates (left panel) and immunoprecipitations (right panel).

ESCRT is a conserved complex in eukaryotes, so we next tested the effects of reduced levels of human ESCRT proteins on human L1 retrotransposition. We chose three human genes (*ALIX*, *CHMP6*, *and CHMP3*) orthologous to yeast ESCRT proteins that 1) had a dramatic effect on Zorro3 retrotransposition in yeast and 2) only have one known human ortholog (Fig 3A). We transfected short interfering RNAs (siRNAs) into HeLa cells, which depleted endogenous ALIX, CHMP6 or CHMP3 protein to levels undetectable by western blot (Fig 3B). Using a standard retrotransposition assay [15,66], these ESCRT-depleted cells were then tested for their ability to support human L1 retrotransposition. In parallel, the cells were also transfected with a control plasmid, pcDNA3, containing the neomycin resistance gene, to determine whether depletion of ESCRT genes limited the ability to form G418 resistant colonies. To take into account possible effects of ESCRT knockdown on cell growth and/or viability, retrotransposition induced G418 resistant colonies were normalized to pcDNA3 induced G418 colonies. Knocking down ALIX, CHMP6, or CHMP3 led to a 67%, 94%, and 81% reduction, respectively, in normalized L1 activity (Fig 3C, S4 Table). ESCRT knockdown does not reduce steady state L1 ORF1p levels (S2 Fig), suggesting that the retrotransposition defects are not due to alteration of L1 expression. To ensure that the reduction in L1 activity was not caused by siRNA off target effects, we designed *ALIX*, *CHMP6*, *and CHMP3* cDNAs with synonomous mutations conferring resistance to their respective siRNA used in Fig 3C. When empty vector was cotransfected in the L1 assay, siRNA knockdown of ALIX, CHMP6, and CHMP3 led to 82%, 95%, and 87% reduction of L1 retrotransposition (Fig 4D, S5 Table), similar to the results in Fig 3C. However, cotransfection of the appropriate siRNA-resistant cDNA in the L1 assay mitigated the effects of ESCRT siRNAs (Fig 3D, S5 Table). In the presence of resistant ALIX cDNA, siALIX reduced relative L1 activity to 54% from 70%, a reduction of only 23%. In the presence of resistant CHMP6 cDNA, siCHMP6 reduced relative L1 activity to 7% from 32%, a reduction of 78%. In the presence of resistant CHMP3 cDNA, siCHMP3 increased relative L1 activity to 69% from 61%. Overexpression of CHMP6 is known to cause cytotoxicity [67], which is consistent with the low levels of G418 colony formation when CHMP6 is overexpressed (Fig 3D, resistant CHMP6 cDNA + scramble siRNA). This makes the results of the CHMP6 rescue ambiguous as compared to the rescue with ALIX and CHMP3 (Fig 3D). In total, our results suggest an important role for ESCRT proteins during human L1 retrotransposition.

### ESCRT/L1 interact via an L1 “late domain”

Some enveloped viruses encode short amino acid motifs called “late domains”, which recruit ESCRT to the budding virus at the plasma membrane [68]. Without ESCRT, viral buds are not pinched off the membrane, and remain attached to the cell [69]. We recognized that the consensus sequence of a known viral late domain, YPX_n_L, corresponds to a motif in human L1 ORF1p (Fig 4A). This motif is conserved among many mammalian L1s (Fig 4B), but the biological function of this L1 motif has not been previously assigned. However, introducing multiple alanine substitutions (YPAKLS->AAAALA) in the motif is known to abolish 99.9% of L1 activity [15]. Although this reduction in retrotransposition activity could be explained, in part, to a disrupted ESCRT/ORF1p interaction, the YPAKLS->AAAALA mutation also leads to poor steady state ORF1p expression and severe defects in L1 RNP formation ([9] and Fig 4C). Thus, the reduction in L1 activity could simply be attributed to the instability of ORF1p and/or the inability to form RNPs.

We took advantage of previous YPX_n_L studies to make a separation of function ORF1p mutant that can still form functional L1 RNPs. The known binding partner of the YPX_n_L late domain is the human protein ALIX, which serves as an adapter protein to recruit ESCRT [69]. The structures of HIV and EIAV YPX_n_L motifs bound to ALIX have been solved and revealed critical interactions required for ALIX/ YPX_n_L binding [70]. The YPX_n_L tyrosine extends into a hydrophobic pocket in ALIX, and mutating this tyrosine eliminates late domain binding. The corresponding Y282 tyrosine within the L1 ORF1p YPX_n_L motif is conserved in mammalian ORF1p, and is present on the external surface of the protein in a position that is presumably accessible for binding to ALIX [11]. We mutated this tyrosine to alanine (Y282A) in L1 ORF1p. In contrast to the YPAKLS->AAAALA mutant, the Y282A mutant produces steady state ORF1p and RNPs indistinguishable from wild type (Fig 4C), suggesting that the Y282A mutant does not alter steady-state levels of L1 RNPs. In addition, we examined RNPs for reverse transcriptase activity using the L1 element amplification protocol (LEAP) assay, the best current biochemical assay for functional L1 RNPs [71]. RNPs isolated from wild type or Y282A transfected cells generated similar LEAP products, whether analyzed by gel electrophoresis or direct DNA sequencing (Fig 4D and S3 Fig). We found no evidence of reverse transcriptase priming upstream of the L1 poly(A) tail (S3 Fig). Thus, based on currently available assays, RNPs produced by a Y282A mutant appear to be functionally “normal”. However, the Y282A mutant exhibited a profound defect (~90% reduction) in retrotransposition (Fig 4E, S6 Table). Co-immunoprecipitation of ALIX with L1 ORF1p was dependent on the YPX_n_L motif, as the Y282A mutant abolished the ORF1/ALIX interaction (Fig 4F). These co-IPs were performed in the presence of RNase A, suggesting that this interaction is not RNA dependent. The strength of the wild type ORF1p/ALIX interaction varied from experiment to experiment (S4 Fig). In sum, our data support the importance of the L1 YPX_n_L late domain for L1/ESCRT interaction and L1 retrotransposition.

### Lysosomal function, glycosylation, and the exocytic pathway are dispensable for L1 retrotransposition

The interaction between ESCRT and L1 proteins strongly suggests that the L1 RNP buds into or interacts with a membrane at some point during L1 replication. Several factors confound our ability to directly pinpoint which membranes, if any, are relevant to L1 biology. L1 retrotransposition is inefficient–even under conditions of massive L1 overexpression over multiple days, typically less than 1% of cells will harbor a retrotransposition event [15,72–75]. In addition, although the L1 mRNA/proteins must enter the nucleus to replicate, most reports suggest that the vast majority of L1 protein is detected in the cytoplasm [18,49,76]. In our hands, we see similar cytoplasmic localization of L1 ORF1p, without obvious co-localization with known cellular organelles (S5 Fig). With the enormous background of L1 particles sequestered in the cytoplasm, it is currently impossible to visually identify the location of the rare “active” RNPs.

**Fig 5:**
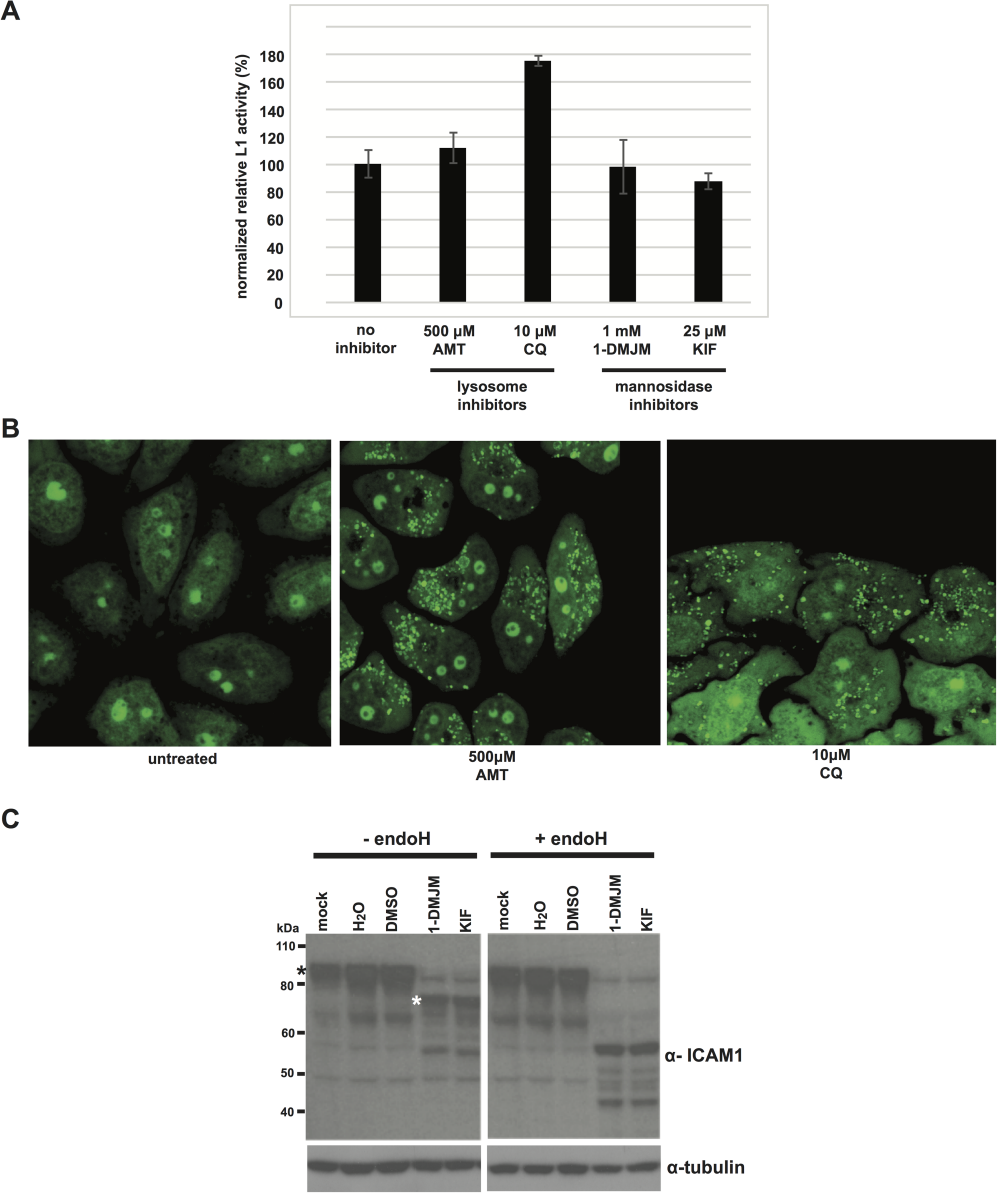
Membrane associated functions dispensible for L1 retrotransposition. **A.** Results of L1 retrotransposition assays. HeLa cells were transfected with pJM101L1.3. 1 hour after transfection, media was replaced with media containing the indicated inhibitors. 24 hours after transfection, cells were selected for G418 resistance. AMT = amantadine. CQ = chloroquine. 1-DMJM = D-mannojirimycin. KIF = kifunensine. **B.** Confocal imaging of inhibitor-treated cells. HeLa cells were treated with the indicated drugs. 24 hours later, cells were stained with acridine orange and visualized by confocal miscroscopy. **C.** Confirmation of glycosidase inhibition. HeLa cells were treated with the indicated inhibitors for 24 hours. The cells were lysed and digested with endoglycosidase H. Shown are western blots of undigested (left panel) and digested (right panel) lysates. Black asterisk = mature glycosylated ICAM-1 form. White asterisk = endoH sensitive ICAM-1 form [106].

We therefore blocked membrane associated functions that we hypothesized could play a role in retrotransposition. ESCRTs are used for budding into endosomes and lysosomes, both of which lead to cargo deposition and degradation in the lysosome. Although it is counterintuitive that lysosome targeting would be required for retrotransposition, the close association of ESCRT with the endosomal membrane system led us to test the requirement of functional lysosomes for L1 activity. We blocked lysosome function using amantadine or chloroquine, drugs that neutralize the acidic lysosome environment [77]**.** L1 activity was unaffected in amantadine treated cells, and increased in chloroquine treated cells (Fig 5A). Cells treated with amantadine or chloroquine demonstrated the previously described swelling of lysosomes when visualized by confocal microscopy [78–80], indicating that the inhibitors were acting as expected (Fig 5B). This suggests that lysosomal function is not required for L1 retrotransposition. We also reasoned that the L1 RNP might be associated with endoplasmic reticulum (ER) membrane. L1 entry into the ER could be required for glycosylation or transport of the L1 RNP. We treated cells with kifunensine or D-mannojirimycin, inhibitors of N-linked glycosylation [81,82]. These drugs reduced glycosylation and production of the mature, endoglycosidase H resistant form of the normally heavily glycosylated ICAM-1 (Fig 5C), indicating an effective glycosylation block. L1 retrotransposition was not significantly affected by this block (Fig 5A). Finally, we treated cells with Exo1 or golgicide A, potent golgi inhibitors that cause golgi collapse and arrest of soluble and membrane associated protein secretion [83,84]. Neither of these drugs had a dramatic effect on L1 retrotransposition frequency (S5 Fig), suggesting that glycosylation or other pathways downstream of the ER are not essential for L1 replication. However, this does not rule out the possibility that L1 RNPs enter the ER for another purpose, such as formation of a shielded vesicular compartment analogous to viral replication compartments [85], or retrograde L1 RNP transport towards the nucleus, the ultimate destination for successful L1 retrotransposition.

### A potential role of ESCRT for L1 nuclear entry

**Fig 6:**
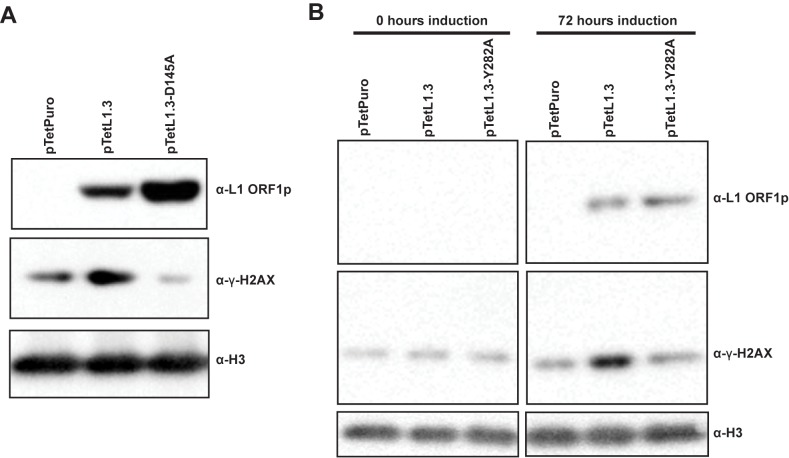
Effect of disrupting the L1/ESCRT interaction of nuclear L1 endonuclease activity. **A.** γH2AX production in response to L1 expression. tet-HeLa cells were transfected with pTetPuro, pTetL1.3, or pTetL1.3-D145A and selected for puromycin resistance. The puromycin resistant populations were treated with doxycycline to induce L1 expression. 72 hours after L1 induction, cell lysates were harvested. Shown are western blots of cell lysates. **B.** γ-H2AX production when ESCRT/ORF1 interaction is disrupted. tet-HeLa cells were transfected with pTetPuro, pTetL1.3, or pTetL1.3-Y282A then treated with doxycycline for 72 hours. Shown are western blots of cell lysates.

Because of the importance of ESCRT in vesicular traffic, and the assumed requirement of L1 RNPs to traffic to the nucleus to replicate, we wondered whether the ESCRT/L1 interaction is necessary for L1 nuclear entry. In our hands, we detect a very low amount of L1 protein in the nucleus when we induce L1 expression (S6 Fig) in tissue culture cells. The small amount of L1 ORF1 that we detect in the nucleus hinders our ability to identify “active” L1 RNPs from the overwhelming amount of background L1 expression. Thus, we chose to use a functional assay as a proxy for nuclear entry of L1 particles. Expression of full-length, replication competent L1 induces the formation of γ-H2AX foci, a marker for DNA double stranded breaks or replication stress [23]. The L1-induced γ-H2AX foci are dependent on L1 endonuclease activity, suggesting that chromosome cleavage by L1 proteins leads to foci formation. As these are nuclear events, we presume that if γ-H2AX foci are formed, enzymatically active L1 particles have entered the nucleus. We used a tetracycline inducible system [86] to induce L1 expression. In this system, full-length L1 is under the control of a Tet-On promoter, allowing L1 expression upon treatment with doxycycline. When we induced L1 expression in tet-HeLa cells, we found L1-dependent upregulation of γ-H2AX by western blot (Fig 6A). This γ-H2AX upregulation was eliminated when we induced expression of L1 with an endonuclease active site mutation (D145A), suggesting that the γ-H2AX upregulation was dependent on L1 endonuclease activity. In a similar manner, we found that when we induce expression of ORF1 Y282A mutant L1, the γ-H2AX upregulation is reduced (Fig 6B). Because the Y282A mutation presumably disrupts the L1/ESCRT interaction, this result is consistent with the hypothesis that the L1/ESCRT interaction is a prerequisite for nuclear L1 endonuclease activity. This further implies that, in the absence of ESCRT, L1 RNPs are impaired for nuclear entry. Alternatively, it is possible that the Y282A mutant blocks endonuclease activity independent of nuclear entry.

## Discussion

Non-LTR retrotransposons have persisted in eukaryotic genomes for hundreds of millions of years, and with few exceptions, are transmitted vertically [1]. This close relationship between retrotransposons and their hosts, and the small number of L1-encoded proteins, suggests that L1 likely exploits host proteins to complete a round of retrotransposition. The two L1-encoded proteins, ORF1p and ORF2p, provide RNA binding, endonuclease, reverse transcriptase, and nucleic acid chaperone activities–these activities are hypothesized to play a role during TPRT. How L1 completes other aspects of its life cycle is still unclear. Although many L1 restriction factors have been discovered [48–50], additional L1 or host encoded activities required for successful retrotransposition are not well understood, with just a handful of these host factors identified. Disruption of the non-homologous end joining (NHEJ) pathway leads to modest decreases in L1 retrotransposition [87], providing genetic evidence suggesting that the NHEJ pathway may assist resolving TPRT intermediates. However, there is no physical evidence for interaction between NHEJ and L1 complexes. In addition, L1 expression generates DNA damage [24,28,88]; hence, it is not surprising that loss of NHEJ in the presence of DNA breaks leads to a decreased colony forming ability. PCNA has recently been found to physically interact with ORF2p via a canonical PCNA-interacting protein (PIP) box motif [49]. The PIP box is required for the ORF2p interaction, and reduction of PCNA expression by RNA interference leads to lower retrotransposition activity, suggesting a positive role for PCNA in L1 retrotransposition. The L1/PCNA interaction requires endonuclease and reverse transcriptase activity, suggesting that the interaction occurs during or after TPRT.

Although significant progress has been made in defining the L1 interactome, the cytoplasmic events leading to active L1 retrotransposition have remained mysterious. The poly(A) binding protein PABPC1 binds L1 RNPs, presumably through the L1 mRNA poly(A) tail [51]. Reduction of PABPC1 by RNA interference results in lower levels of functional L1 RNPs and reduced retrotransposition. This is consistent with the requirement of a poly(A) tail for L1 retrotransposition [89]. Once made, how a functional L1 RNP traffics to the nucleus to initiate TPRT is not known. L1 RNPs are localized in a diffuse pattern with discrete cytoplasmic foci when visualized by immunofluorescence [18,49,76,90]. The cytoplasmic foci colocalize with stress granule markers. However, even under conditions of massive overexpression and production of L1-containing stress granules, retrotransposition frequency is low (<1% in colony forming retrotransposition assays) and nuclear localization of L1 RNPs is rare. Stress granules are degraded by autophagy [91], and disruption of genes critical for autophagy result in increased L1 expression and retrotransposition activity [92]. Together, this evidence suggests that L1 cytoplasmic foci may simply be RNPs that are sequestered for degradation. The rare L1 RNPs that are trafficking to the nucleus may be impossible to monitor with current methodology.

In this study, we provide evidence that the ESCRT complex interacts with LINE RNPs and is critical for robust LINE retrotransposition, including human L1 retrotransposition. The L1 ORF1p/ALIX interaction depends on an ORF1p YPX_n_L late domain. In viruses, YPX_n_L motifs have been shown to directly bind with ALIX [69, 70], suggesting that L1 ORF1p also directly interacts with ALIX via this motif. The established role of ESCRT in membrane budding/fusion suggests that active L1 RNPs may be intimately associated with membranes at some point during a successful retrotransposition event. Membrane association of LINE RNPs is a relatively new concept first suggested by the discovery of esterase domains encoded by the ORF1p of some members of the CR1 non-LTR retrotransposon clade [93]. These esterase domains are classified as lipolytic acetylhydrolases, and more recently the esterase domain of the purified zebrafish LINE element ZfL2-1 ORF1p was shown to be enzymatically active and able to bind phospholipids and liposomes *in vitro* [94]. An N-terminally truncated version of purified human ORF1p (which lacks an identifiable esterase domain) also co-migrated with lipids in a lipid floatation assay, suggesting that membrane interaction may be universally conserved for LINEs [94]

**Fig 7:**
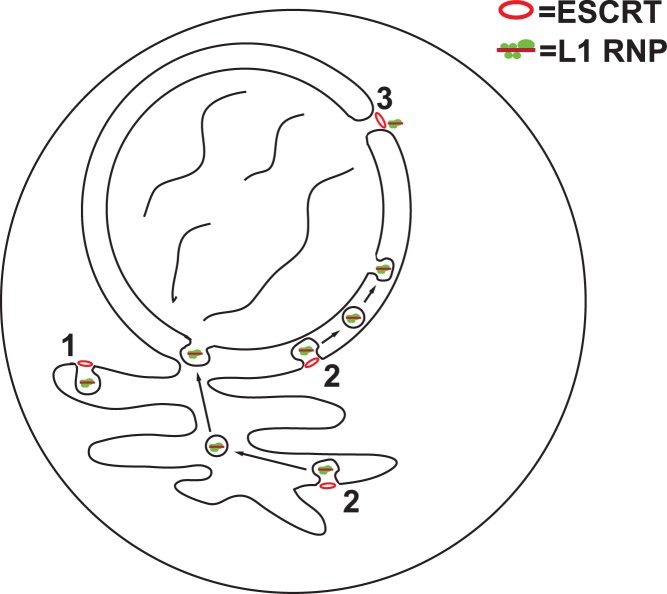
Speculative models for how ESCRT assists L1 replication. 1) Formation of a membrane bound compartment for RNP maturation. 2) Budding into the endoplasmic reticulum or outer nuclear membrane in transit to the nucleus. 3) Recruitment of L1 RNPs during nuclear envelope reformation.

Although we do not have the ability to visually distinguish L1 RNPs that are in the process of retrotransposition from the predominant background of sequestered L1 RNPs, we can speculate how ESCRT assists L1 replication based on this study, combined with current knowledge. The rarity of horizontal transmission of non-LTR retrotransposons suggests that normal L1 replication does not involve functional L1 RNPs leaving the cell to “infect” other cells (and/or species). Thus, we feel it is unlikely that ESCRT functions to release L1 particles from the plasma membrane. Our data also suggest that membrane trafficking related processes such as glycosylation, secretion, and lysosomal degradation are also dispensable for L1 retrotransposition. When we disrupt the ESCRT/L1 interaction, L1 endonuclease activity in the nucleus is reduced. Although this reduction in endonuclease activity in the nucleus suggests an impairment in the nuclear entry of L1 RNPs, it is also possible that L1 RNPs can enter the nucleus without ESCRT, but are unable to cut DNA. Plausible models for the role of ESCRT during L1 retrotransposition are that ESCRT is involved in the formation of a membrane bound compartment for RNP maturation, akin to the ESCRT-dependent replication compartments of some viruses [85], or ESCRT helps active L1 RNPs traffic to the nucleus by enabling the RNPs to cross a membrane barrier (Fig 7). For example, L1 RNPs could bud into the outer nuclear membrane, or the contiguous endoplasmic reticulum, to form perinuclear or intracisternal vesicles. Fusion of these vesicles with the inner nuclear membrane could deposit L1 RNPs into the nucleus. Although this would be an unusual mechanism for nuclear entry, a similar ESCRT-dependent process in the opposite direction, called nuclear egress, is used by herpesviruses and endogenous RNPs to exit the nucleus [95,96]. In addition, nuclear envelope reformation after mitosis is an ESCRT-dependent process that is topologically equivalent to membrane bud neck constriction [63,64]. Recruitment of L1 RNPs to the nuclear membrane by ESCRT after mitosis could be a route for L1 nuclear entry. Finally, ESCRT could assist L1 retrotransposition in a non-membrane dependent manner. However, this would require a currently undefined function of ESCRT. Distinguishing between these various models will require technological advances in tracking the cellular location of actively retrotransposing L1 particles.

## Methods

### Yeast strains

The yeast knockout collection [53] was obtained from the ATCC (GSA-5) and was in the BY4741 background (MATa his3Δ1; leu2Δ0; met15Δ0; ura3Δ0) [97]. Construction of strains JHY146 (MATα his3Δ200 ura3-167 GAL+ lys2::mHIS3AI) and JHY148 (MATα his3Δ200 ura3-167 GAL+ lys2::Zorro3mHIS3AI) have been described previously [52].

### Cell culture

HeLa cells were a gift from John Moran [15]. Tet-HeLa cells were from Clontech (Mountain View, CA). Tet-293 cells were a gift from Jef Boeke [51]. All cells were grown in Dulbecco’s Modified Eagle Media (Invitrogen #11965118) supplemented with 10% fetal bovine serum (Invitrogen, #16000069) and 1% Penicillin/Streptomycin (Invitrogen, #15140122). Puromycin resistant plasmids were selected with 1 μg/mL puromycin (Invivogen #ant-pr-1) and maintained with 0.25 μg/mL puromycin. Tet-inducible proteins were induced with 500 ng/mL doxycycline (Fisher Scientific #BP2653). Cells were grown at 37°C unless otherwise specified.

### Oligonucleotides

Oligonucleotides used in this study are listed in S9 Table.

### Plasmids

All cloned PCR and site directed mutagenesis products described below were completely sequenced. Sequence files of all plasmids are available upon request.

p425Zorro3mMET15AI (Zorro3 retrotransposition plasmid)—The yeast MET15AI retrotransposition marker was made by first PCR amplifying the MET15 gene from the yeast strain BY4742 using primer JH308/JH311, then TOPO cloning the amplicon into pCR2.1 to make pCRMET15. An artificial intron was amplified from pSCZorro3mHIS3AI [52] using the primers JH251/JH255, digested with PvuII/SnaBI, and cloned into the AleI site of pCRMET15 to make pCRMET15AI. The MET15AI marker was removed from pCRMET15AI with XhoI, blunted with T4 DNA polymerase/dNTPs, and cloned into the T4 DNA polymerase blunted SalI site of pSCZorro3 [52] to make pSCZorro3mMET15AI. Oligonucleotides JH28/JH29 were annealed then digested with RsaI/SacI. pRS425 [98] was digested with XhoI, blunted with T4 DNA polymerase, then further digested with SacI. Oligonucleotides JH28/JH29 were annealed then digested with RsaI/SacI, then cloned into the digested pRS425 vector to make pRS425FE. The EagI/FseI GAL1-Zorro3mMET15AI fragment from pSCZorro3mMET15AI was cloned into the EagI/FseI of pRS425FE to make p425Zorro3mMET15AI. Standard site directed mutagenesis techniques were used to make p425Zorro3RTmutmMET15AI, which is identical to p425Zorro3mMET15AI but with D674A, D675A missense mutations in ORF2p.

p415GAL-lacZ was made by cloning the SmaI/HindIII lacZ fragment from pCMV-lacZ (Clontech) into the SmaI/HindIII sites of p415Gal1 [99].

pGAL-HASNF7, pGAL-HAVPS4, pGAL-HAVPS23, pGAL-HABRO1, pGAL-HASNF8, pGAL-HAVPS20 –These plasmids are based on the p426GAL1 vector [99], with a URA3 selection marker, 2 micron replication origin and GAL1 promoter. Using standard PCR cloning procedures, a 3x HA tag was inserted, along with each ORF amplified from BY4741 genomic DNA.

pJM101L1.3, pJM105L1.3, and pJM110 were gifts from John Moran and Haig Kazazian [15,66,100]. These plasmids were derived from a pCEP4 backbone (Invitrogen).

pJM101L1.3Y282A was made site directed mutagenesis of pJM101L1.3 using standard cloning techniques.

pTetPuro was made by digesting pMT302 (gift from Jef Boeke [49]) with NotI/BstZ17I, blunting, and religation.

pTetL1.3 was made by ligating a NotI/BstZ17I fragment from pJM101L1.3 into the NotI/BstZ17I sites of pMT302.

pTetL1.3–282 was made by ligating a NotI/BstZ17I fragment from pJM101L1.3Y282A into the NotI/BstZ17I sites of pMT302.

pTetL1.3–145 was made by ligating a PmlI/BbvCI fragment from pQF288 (a gift from Jef Boeke [16]) and a BbvCI/BamHI fragment from pTetL.13 into the PmlI/BamHI sites of pTetL1.3.

pEF was made by digesting pTracerEF A (Invitrogen) with SphI/BspQI, blunting, and religating.

pEFALIX-2HA was made by filling in annealed JH-IM54/JH-IM55 by PCR, then amplifying this product with JH-IM56/JH-IM57 to make tandem HA tags. ALIX cDNA was amplified from mammalian gene collection clone MGC-17003 using primers JH-IM50/JH-IM61. The amplified ALIX cDNA and tandem HA tags were fused together in a PCR with JH-IM50/JH-IM57. The product was digested with EcoRI and cloned into EcoRI/EcoRV digested pEF. Although this strategy intended to add 3 HA tags, in practice it added 2 HA tags. cDNA expression is under the control of the strong constitutive elongation factor-1α promoter.

pEFCHMP6-2HA was made by filling in annealed JH-IM54/JH-IM55 by PCR, then amplifying this product with JH-IM56/JH-IM57 to make tandem HA tags. CHMP6 cDNA was amplified from mammalian gene collection clone MGC-19477 using primers JH-IM44/JH-IM58. The amplified CHMP6 cDNA and tandem HA tags were fused together in a PCR with JH-IM44/JH-IM57. The product was digested with MfeI/XbaI and cloned into EcoRI/XbaI digested pEF.

pEFCHMP3-2HA was made by filling in annealed JH-IM54/JH-IM55 by PCR, then amplifying this product with JH-IM56/JH-IM57 to make tandem HA tags. CHMP3 cDNA was amplified from mammalian gene collection clone MGC-11028 using primers JH-IM53/JH-IM59. The amplified CHMP3 cDNA and tandem HA tags were fused together in a PCR with JH-IM53/JH-IM57. The product was digested with MfeI/XbaI and cloned into EcoRI/XbaI digested pEF.

pEFALIXs19465mut (cDNA resistant to the s19465 siRNA) was made by amplifying the ALIX cDNA from MGC-17003 using a series of fusion PCRs with primer sets JH-IM50/JH-IM88 and JH-IM87/JH-IM62, digesting with EcoRI/EagI, and cloning into the EcoRI/EagI sites of pEF.

pEFCHMP6s28474mut (cDNA resistant to the s28474 siRNA) was made by amplifying the CHMP6 cDNA from MGC-19477 using a series of fusion PCRs with primer sets JH-IM77/JH-IM81 and JH-IM74/JH-IM67, digesting with EcoRI/XbaI, and cloning into the EcoRI/XbaI sites of pEF.

pEFCHMP3s35990mut (cDNA resistant to the s35990 siRNA) was made by amplifying the CHMP3 cDNA from MGC-11028 using a series of fusion PCRs with primer sets JH-IM78/JH-IM82 and JH-IM75/JH-IM68, digesting with MfeI/XbaI, and cloning into the EcoRI/XbaI sites of pEF.

pGEX6p2Z3ORF1 –Zorro3 ORF1 was amplified with primers JH256/JH257, digested with BamHI/SalI, and ligated into the BamHI/XhoI sites of pGEX-6P-2 (GE Healthcare).

pGEX-3’hORF1 –synthetic human L1 [101] was amplified with primer JH1004/JH1005, digested with EcoRI/BamHI, and ligated into the EcoRI/BamHI sites of pGEX-6P-2.

### Antibodies

G01 (rabbit polyclonal anti-Zorro3 ORF1)–The plasmid pGEX6p2Z3ORF1 was transformed into BL(DE3)plysS competent bacteria and GST-Z3ORF1p expression was induced with IPTG. GSTZ3ORF1p was purified with a glutathione sepharose column, followed by FPLC on a Superose 6 10/300 GL column. Fractions of the highest purity and concentration were sent to Proteintech (Chicago, IL) for antibody production. Characterization of G01 is shown in S7 Fig.

JH73 and JH74 (rabbit monoclonal anti-L1 ORF1)–The plasmid pGEX-3’hORF1 was transformed into BL(DE3)plysS bacteria and GST-3’hORF1 was purified as described for GSTZ3ORF1p. Purified GST-3’hORF1 was sent to Epitomics (now Abcam) for rabbit monoclonal antibody production. Hybridoma supernatants were screened by western blot, immunoprecipitation, immunofluorescence, and immunohistochemistry of paraffin embedded samples for the best clones for all applications. Antibody was purified from hybridoma supernatant with Protein A Agarose (Pierce). Characterization of JH73 and JH74 is shown in S8 Fig. Additional characterization is shown in [49].

Commercially available antibodies used: anti-HA affinity matrix (Sigma #11815016001), mouse anti-HA clone 12CA5 (Sigma #11583816001), rat anti-HA clones 3F10 (Sigma #11867423001), rabbit polyclonal anti-ALIX (Bethyl #A302-938A), mouse monoclonal anti-CHMP3 clone F-1 (Santa Cruz #sc-166361), rabbit polyclonal anti-CHMP6 clone FL-201 (Santa Cruz #sc-67231), rabbit polyclonal anti-H3 (Abcam #ab1791), mouse monoclonal anti-γ-H2AX clone JBW301 (EMD Millipore #05-636-I), mouse monoclonal anti-ICAM-1, clone 15.2 (Santa Cruz #sc-107), mouse monoclonal anti-tubulin clone DM1A (Sigma #T6199), rabbit monoclonal anti-EEA1 clone C45B10 (Cell Signaling Technology #3288), rabbit monoclonal anti-Rab7 clone D95F2 (Cell Signaling Technology #9367), mouse monoclonal anti-LAMP2 clone H4B4 (Abcam #ab25631), mouse monoclonal anti-calnexin clone AF18 (ThermoFisher #MA3-027), rabbit monoclonal anti-AIF clone D39D2 (Cell Signaling Technology #5318), mouse monoclonal anti-RCAS1 clone D9 (Santa Cruz #sc-398052), rabbit polyclonal anti-EDC4 (Cell Signaling Technology #2548), rabbit monoclonal anti-eIF3H clone DC91 (Cell Signaling Technology #3413).

### Screen for Zorro3 host factors

Lithium acetate transformation was used to transform all strains of the yeast knockout collection with the plasmid p425Zorro3mMET15AI. For controls, BY4741 was transformed with p425Zorro3mMET15AI or p425Zorro3RTmutmMET15AI. Transformants were selected on SC-leu plates. Three clones from each transformation were used to make 1.5 cm^2^ patches on SC-leu+galactose plates. After 6 days at 23°C, the patches were replica plated to SC-met plates. Strains that exhibited a qualitatively noticeable decrease in Zorro3 retrotransposition were selected for quantitative Zorro3 retrotransposition assays [52]. Briefly, transformants were inoculated in 3 mL SC-leu cultures and incubated for 3 days at 23°C on a roller drum. After incubation, 200 μL of each culture was plated on an SC-met plate, and 10 μL of a 1:1000 dilution was plated on a YPD plate. Retrotransposition frequency was calculated as (SC-met colonies)/(YPD colonies x dilution factor).

### β-galactosidase assay of yeast lysates

Yeast strains were transformed with p415GAL-lacZ and selected on SC-leu plates. The transformed strains were grown overnight in SC-leu media with glucose, washed with H_2_O, then resuspended and incubated overnight in SC-leu with galactose. Cell concentrations of cultures were normalized to a constant OD_600_. 1 mL of each normalized culture was used to assay for β-galactosidase activity using a previous described method [102]. Briefly, the pelleted culture was resuspended in 500 μL of Z buffer. 50 μL of 0.1% SDS and 50 μL chloroform were added and the solution was vortexed. 100 μL of a 4 mg/mL stock of o-nitrophenol-β-D-galactoside (ONPG) was added and incubated at 37 degrees for 15 minutes. The reaction was quenched with 500 μL of 1 M Na_2_CO_3_. 100 uL of each supernantant was transferred into a 96 well plate, and OD_420_ was measured with a plate reader. Wild-type BY4741 transformed with p415GAL-lacZ and grown in glucose was used as to measure background activity and was subtracted from all other values. β-galactosidase activity of BY4741 transformed with p415GAL-lacZ was set to 1, and for each experiment, the activity of knockout strains were normalized to this value.

### Immunoprecipitations from yeast

Strains JHY146 or JHY148 were transformed with pGAL-HASNF7, pGAL-HAVPS4, pGAL-HAVPS23, pGAL-HABRO1, pGAL-HASNF8, or pGAL-HAVPS20 and selected on SC-ura plates. Zorro3 and the respective ESCRT proteins were induced in SC-ura+galactose cultures for 16 hours, and lysed in a BioSpec mini bead beater with 25 mM Tris-Cl pH 7.4, 150 mM NaCl, 0.5% Triton X-100, 0.5% Na-deoxycholate, 1 mM EDTA, 4% glycerol, 1% protease inhibitor cocktail (Sigma #P2714), 1 mM PMSF, 10 mM MgCl2, and acid washed glass beads. Where indicated, 0.5 μg/mL RNase A was added to the IPs. Antibody G01 was used for IP and western blot of Zorro3 ORF1p, anti-HA affinity matrix was used for HA IP, and anti-HA clones 12CA5 or 3F10 were used for HA western blot.

### L1 retrotransposition assays

To knockdown expression of ESCRT proteins and test L1 retrotransposition, we modified an established retrotransposition assay [66]. 2 x 10^5^ HeLa cells/well were seeded in 6-well plates. 24 hours after seeding, cells were transfected with 1 μg of L1 reporter plasmid (pJM101L1.3 or pJM105L1.3) and 15 pmol of silencer select siRNA (Ambion—s19465 for ALIX, s29474 for CHMP6, s35990 for CHMP3, or scramble negative control) using Lipofectamine 2000 (Invitrogen) according to the manufacturers protocol. 24 hours post-transfection, G418 selection (600 μg/mL) was started and continued for 11–12 days. G418-resistant colonies were fixed and stained with 250 μl/well of modified Giemsa stain (Sigma #GS500). To assess whether knockdown affected transfection efficiency or viability, we performed parallel co-transfections with 10 ng pcDNA3 (Invitrogen) in place of the L1 reporter plasmid. G418-resistant colonies from the retrotransposition assay were normalized to the respective pcDNA3 colony forming assays to give “normalized L1 activity”. For siRNA rescue retrotransposition experiments, co-transfections were performed as described above with the following amounts of nucleic acids: 0.5 μg L1 reporter construct, 0.5 μg siRNA resistant cDNA or empty vector, and 15 pmol siRNA.

To test the effectiveness of siRNA knockdown, we performed separate experiments where we co-transfected HeLas with pEFALIX-2HA, pEFCHMP6-2HA, or pEFCHMP3-2HA and the relevant siRNA or scramble negative control siRNA. Knockdown was assessed by western blot with anti-HA 12CA5 antibody.

To perform standard L1 retrotransposition assays (in the absence of siRNA knockdown), 2.5 x 10^5^ HeLas/well were seeded in 6-well plates. 24 hours after seeding, cells were transfected with 1 μg L1 reporter plasmid and 3 μL XtremeGENE9 (Roche) according to the manufacturer’s protocol. 24 hours post-transfection, G418 selection (600 μg/mL) was started and continued for 11–12 days. G418-resistant colonies were fixed and stained with 250 μl/well of modified Giemsa stain (Sigma #GS500). To assess transfection efficiency, extra wells were set aside for each condition and analyzed for ORF1p expression by western blot. In cases where inhibitors were added to the retrotransposition assays, the inhibitors were added to the media 1 hour post-transfection. The final concentration of drugs used were as follows: 500 μM amantadine (Sigma #A1260), 10 μM chloroquine (Sigma #C6628), 25 μM kifunensine (Sigma #K1140), 1 mM D-mannojirimycin (Sigma #D9160), 2.5 μM golgicide (Sigma #G0923), 75 μM Exo1 (Sigma #E8280).

### L1 RNP isolation and LEAP assays

RNP isolation and LEAP assays were based on previous published work [71]. Briefly, to isolate L1 RNPs, 2 x 10^6^ HeLa cells were plated in a T75 cell culture flask. Cells were transfected 24 hours later with 20 μg of L1 plasmid per flask. Three days post-transfection, medium was replaced by DMEM supplemented with 0.2 mg/mL hygromycin B and exchanged daily. On day 7 post-transfection, one T-75 flask was seeded with 2 x 10^6^ untransfected HeLa cells to be used as a negative control. On day 10 post-transfection, transfected and untransfected cells were harvested and whole-cell lysates were prepared and subjected to ultracentrifugation as described previously [71].

The LEAP reaction was performed as described previously [71] with the following changes: a 1 μL aliquot of the pelleted samples was incubated with 50 mM Tris-HCl (pH 7.5), 50 mM KCl, 5 mM MgCl_2_, 10 mM DTT, 0.4 mM LEAP primer, 20 U RNaseOut (Invitrogen), 0.2 mM dNTPs and 0.05% Tween 20 in a final volume of 50 μL for 1 hour at 37°C. After incubation, 1 μL of LEAP reaction was used in a standard 30 μL PCR using AmpliTaq DNA polymerase (Applied Biosystems) and 0.4 mM of each linker PCR primer and L1.3 end primer according to the manufacturer’s protocol. PCR conditions as previously described [71]. The complete reaction was visualized on a 2% agarose gel with Ethidium Bromide.

### Immunoprecipitations from human cells

Tet-293 cells were transfected with pTetPuro, pTetL1.3, or pTetL1.3–282. 24 hours post-transfection, cells were selected with puromycin. 100 mm plates of selected cells were transfected with 21 μg of pEFALIX-2HA in N,N-bis(2-hydroxyethyl)-2-amino-ethanesulfonic acid (BES) buffer as previously described [103]. L1 expression was induced with doxycycline, and after 48 hours of induction cells were lysed using a Precellys 24 homogenizer (Bertin Instruments) and acid-washed beads (Sigma #G8772) in 25 mM HEPES pH 7.4, 200 mM NaCl, 1 mM MgCl_2_, 1 mM CaCl_2_, 1 x protease inhibitor cocktail (Sigma #S8830), 40 units DNase I, 2.5 mg RNaseA, and 10% glycerol. L1 ORF1p was immunoprecipitated with JH74. Western blot analysis was performed with JH73 (ORF1p) and anti-ALIX or 12CA5 (HA).

### Confocal microscopy

HeLa cells were seeded at 2 x 10^5^ cells/dish in 35 mm glass bottom dishes (MatTek #P35G-0-10-C). 24 hours after seeding, cells were treated with 10 μM chloroquine or 50 μM amantadine. After 24 hours, fresh media/drug was added along with 1 μM acridine orange, and incubated at 37°C for 1 hour. The media was washed and replace with PBS, then directly imaged on an Olympus FV1000 confocal miscroscope with a FITC filter.

### L1 and γ-H2AX induction

Tet-HeLa cells were transfected with pTetPuro, pTetL1.3, pTetL1.3–282, or pTetL1.3–145 and transfected populations were selected with puromycin. In selected cells, L1 expression was induced with doxycycline, and whole cell lysates were analysed by western blot for H3 and γ-H2AX at 72 hours.

### Immunofluorescence

10,000 cells containing the plasmid pTetL1.3 were seeded on coverslips treated with 0.01% poly-L-lysine in 24-well plate. Approximatively 16 hours after seeding, L1.3 expression was induced with 0.5 ug/ml doxycycline. Cells were fixed 48 hours after induction with 4% paraformaldehyde in PBS pH 7.4 for 15 minutes at room temperature (RT). After fixation, cells were washed 3 times with PBS pH7 .4. For calnexin, RCAS1, EEA1, RAB7 and AIF staining, cells were permeabilized with PBS pH 7.4 containing 0.1% Triton X-100 for 15 minutes at RT. Nonspecific interactions were blocked with 10% normal donkey serum (NDS) in PBS for 1 hour at RT. Staining with primary antibody, diluted in PBS pH 7.4 and 2% NDS was done overnight at 4°C. The following dilutions for primary antibody were used: anti-ORF1p clone 4H1 (mouse monoclonal antibody) 0.75 μg/ml, anti-ORF1p clone JH74 (rabbit monoclonal antibody) 0.15 μg/ml; anti-calnexin 1:1000; anti-RCAS1 1:200; anti-EEA1 1:200; anti-RAB7 1:100; anti-AIF 1:400. After primary antibody incubation, cells were washed 3 times with PBS pH 7.4. Secondary antibodies were used at 1:1000 dilution in PBS pH7.4 with 2% NDS for 1–2 hours at RT. To detect anti-ORF1p antibodies, we used Alexa Fluor 488 Donkey anti-Mouse IgG (ThermoFisher # A21202) or Alexa Fluor 488 Donkey anti-Rabbit IgG (ThermoFisher # A21206). To detect antibodies against organelle markers, we used Alexa Fluor 594 Donkey anti-Mouse IgG (ThermoFisher # A21203) or Alexa Fluor 594 Donkey anti-Rabbit IgG (ThermoFisher #A21207). After incubation with secondary antibody, cells were washed 2 times with PBS pH 7.4, incubated with 1 μg/ml DAPI in PBS pH 7.4 for 5 minutes and washed once with PBS pH 7.4. Coverslips were mounted on glass slides with ProLong Gold antifade reagent (Life Technologies #P36930). For LAMP2 and eIF3h staining, cells were incubated with PBS pH 7.4 with 0.3% Triton X-100 and 5% NDS for 1 hour at RT to permeabilize cells and block nonspecific protein interactions. Staining with primary antibody was done overnight at 4°C in PBS pH 7.4 with 0.3% Triton X-100 and 1% BSA. Anti-LAMP2 antibody was used at a 1:100 dilution and anti-eIF3h antibody at a 1:400 dilution. Cells were washed 3 times with PBS pH 7.4 and incubated with secondary antibodies diluted to 1:500 in PBS pH 7.4 with 0.3% Triton X-100 and 1% BSA for 1–2 hours at RT. After secondary antibody, cells were washed, DAPI stainied and mounted as described above.

## Supporting information

S1 Fig RNA independence of Zorro3/ESCRT interaction. JHY148 was transformed with empty vector (pGAL-HA) or plasmids encoding the indicated HA-tagged cDNAs. After galactose induction to express Zorro3 and the transformed cDNAs, cells were lysed and immunoprecipitations performed with anti-Zorro3 ORF1p antibody, with or without 0.5 μg/mL RNase A. Whole lysates (left panels) or immunoprecipitations (right panel) were separated by SDS-PAGE and subjected to western blot with the indicated antibodies.
(EPS)

S2 Fig Effect of ESCRT knockdown on steady state L1 ORF1p levels. tet-Hela cells were transfected with episomally replicating pTetL1.3 or pTetPuro (empty vector) plasmids, and the plasmids were selected with puromycin. The puromycin-selected cell lines were transfected with the indicated siRNAs, then treated with doxycycline to induce expression of L1. Shown are western blots of cell lysates harvested 48 hours after L1 induction. Anti-histone H3 serves as a loading control.
(EPS)

S3 Fig TA clones from LEAP assays. Shown are TA-cloned LEAP products from pJM101L1.3 or pJM101L1.3-Y282A.
(PDF)

S4 Fig Varying strength of ORF1p/ALIX interaction. Shown are five independent co-immunoprecipitations of L1 ORF1p and ALIX. The top leftmost panel is the same experiment shown in Fig 4F.
(EPS)

S5 Fig Retrotransposition in the presence of golgi inhibitors. **A**. Results of L1 retrotransposition assays. HeLa cells were transfected with pJM101L1.3. 1 hour after transfection, media was replaced with media containing the indicated inhibitors. 24 hours after transfection, cells were selected for G418 resistance. DMSO = dimethylsulfoxide. DMF = dimethylformamide. Data normalized for drug toxicity as shown in S8 Table. **B.** Confirmation of golgi inhibition. HeLa cells were treated with the indicated inhibitors for 24 hours. The cells were lysed and digested with endoglycosidase H. Shown are western blots of undigested (left panel) and digested (right panel) lysates. Inhibition of golgi formation is expected to reduce mature glycosylation of ICAM-1. Black asterisk = mature glycosylated ICAM-1 form. White asterisk = endoH sensitive ICAM-1 form.
(TIFF)

S6 Fig Indirect immunofluorescence of L1 ORF1p in tet-HeLa cells. Tet-HeLa cells selected for pTetL1.3 plasmid were treated with 0.5 μg/mL doxycycline for 48 hours to induce L1 expression. At 48 hours, cells were fixed and stained with antibodies against L1 ORF1p and the indicated cellular markers. Alexa Fluor 488 secondary antibodies were used to detect anti-ORF1 antibodies, and Alexa Fluor 594 secondary antibodies were used to detect the cellular markers. DAPI staining shows the nucleus. Shown are representative single planes of confocal imaging. The anti-ORF1p antibody used (JH74 or 4H1) for each subfigure depends on the species source of the cell marker antibody. **A.** anti-ORF1p and anti-EEA1 (marker for early endosomes). **B.** anti-ORF1p and anti-RAB7 (marker for late endosomes). **C.** anti-ORF1 and anti-LAMP2 (marker for lysosomes). **D.** anti-ORF1 and anti-calnexin (marker for endoplasmic reticulum). **E.** anti-ORF1 and anti-AIF (marker for mitochondira). **F.** anti-ORF1 and anti-RCAS1 (marker for golgi). **G.** anti-ORF1 and anti-EDC4 (marker for P bodies). **H.** anti-ORF1 and anti-eIF3H (marker for stress granules).
(TIFF)

S7 Fig Characterization of anti-Zorro3 ORF1p G01 antibody. **A.** Coomassie-stained gel of purified GST-ORF1p. pGEX6p2Z3ORF1 was transformed into BL(DE3)plysS bacteria and GST-Z3ORF1p expression was induced with IPTG. GSTZ3ORF1p was purified with a glutathione sepharose column, followed by FPLC on a Superose 6 10/300 GL column. Shown is the fraction sent for antibody production. Grey arrow indicated GST-ORF1p. **B.** Western blot of lysates from yeast expressing galactose induced ORF1p (left) or galactose-induced 3xHAORF1 (right panel). Addition of the 3xHA tag shifts ORF1 upward.
(EPS)

S8 Fig Characterization of anti-L1 ORF1p antibodies JH73 and JH74. **A.** Western blots. Tet-HeLa cells were transfected with pTetPuro or pTetL1.3 and selected with puromycin. Puromycin selected pools were treated with doxycycline for 48 hours. Shown are western blots of whole cell lysates harvested at 48 hours. **B.** Immunoprepcipitations. Tet-HeLa cells were transfected with pTetPuro or pTetL1.3 and selected with puromycin. Puromycin selected pools were treated with doxycycline for 48 hours. Cell lysates taken at 48 hours were immunoprecipitated with JH73 or JH74, then western blotted with JH73. **C.** Immunohistochemistry. Tet-HeLa cells transfected with pTetL1.3 were treated with or without doxycycline. 48 hours after treatment, cells were fixed with 4% paraformaldehyde, paraffin embedded, and subjected to immunohistochemistry with Vectastain ABD HRP kit and 3,3’-diaminobenzadine tetrahydrochloride (Vector Laboratories).
(TIFF)

S1 Table Strains reduced for Zorro3 retrotransposition–assay results. (XLSX)

S2 Table Zorro3 retrotransposition assay results in ESCRT knockout strains. (XLSX)

S3 Table β-Galactoside assay in selected knockout strains transformed with p415GAL-lacZ. The indicated strains were transformed with p415GAL-lacZ and induced with galactose to express β-galactosidase. Uninduced (wt BY4741 strain grown in glucose) β-galactosidase activity was subtracted from all readings to account for background. Background corrected β-galactosidase activity in knockout strains were normalized to background corrected β-galactosidase activity in wt BY4741 strains. Strains with a null ESCRT gene are highlighted in yellow. If tested, the quantitative retrotransposition frequency for each strain is listed. The retrotransposition frequencies are taken from S1 Table, sheets 3–13.
(XLSX)

S4 Table L1 retrotransposition assay results in ESCRT knockdown HeLa cells. Data used to make Fig 3C. The numbers used for the histogram in Fig 3C are highlighted in yellow.
(XLSX)

S5 Table L1 retrotransposition rescue with siRNA assay results. Data used to make Fig 3D. The numbers used for the histograms in Fig 3D are highlighted in yellow.
(XLSX)

S6 Table L1 retrotransposition assay results Y282A mutant. Data used to make Fig 4E. The numbers used for the histogram in Fig 4E are highlighted in yellow.
(XLSX)

S7 Table L1 retrotransposition assays with lysosomal or glycosidase inhibitors. Data used to make Fig 5A. The numbers used for the histogram in Fig 5A are highlighted in yellow.
(XLSX)

S8 Table L1 retrotransposition assays with golgi inhibitors. Data used to make S5 Fig. The numbers used for the histogram in S5A Fig are highlighted in yellow.
(XLSX)

S9 Table Oligonucleotides used in this study. (XLSX)
