# Supplementary material for: A conserved role for the ESCRT membrane budding complex in LINE retrotransposition
Source: PLoS Genet. 2017 Jun 6;13(6):e1006837. doi: 10.1371/journal.pgen.1006837 (PMC5478143; doi:10.1371/journal.pgen.1006837)
Supplement: S3 Fig — Shown are TA-cloned LEAP products from pJM101L1.3 or pJM101L1.3-Y282A. (PDF) [file pgen.1006837.s003.pdf]

S3 Figure

Sequence alignment of LEAP TA clones

L1.3

Clone	Sequence
2-7	ATGATAAGATACATTGATGAGTTTGGACAAACCACAACTAGAATGCAGTGAAAAAAATGCTTTATTTGTGAAATTTGTGATGCTATTGCTTTATTTGTAACCATTATAAGCTGCAATAAACAAGTTAACAAC(A)$_{156}$
2-9	......................C...(A)$_{136}$
2-21	....(A)$_{133}$
2-23	....(A)$_{108}$
2-15	....(A)$_{101}$
2-5	....(A)$_{98}$
2-3	..........G...(A)$_{94}$
2-10	....(A)$_{93}$
2-8	....(A)$_{91}$
2-2	....(A)$_{82}$
2-4	....(A)$_{81}$
2-6	..NN..(A)$_{72}$
2-13	....(A)$_{70}$
2-20	....(A)$_{69}$
2-11	....(A)$_{65}$
2-22	....(A)$_{59}$
2-16	....(A)$_{59}$
2-1	....(A)$_{44}$
2-14	....................................G.............G..(A)$_{12}$

n = 19

L1.3-Y282A

Clone	Sequence
4-13	ATGATAAGATACATTGATGAGTTTGGACAAACCACAACTAGAATGCAGTGAAAAAAATGCTTTATTTGTGAAATTTGTGATGCTATTGCTTTATTTGTAACCATTATAAGCTGCAATAAACAAGTTAACAAC(A)$_{111}$
4-5	....(A)$_{110}$
4-1	....(A)$_{100}$
4-24	....(A)$_{89}$
4-9	...............A...T..................AAC(A)$_{86}$
4-20	....(A)$_{80}$
4-12	....(A)$_{80}$
4-19	....C......................................(A)$_{69}$
4-25	....(A)$_{60}$
4-16	....(A)$_{58}$
4-4	....(A)$_{58}$
4-10	............G...(A)$_{57}$
4-14	.......A...C..(A)$_{57}$
4-15	....(A)$_{50}$
4-11	....AAC(A)$_{46}$

n = 15
