# Supplementary figures and images for: A conserved role for the ESCRT membrane budding complex in LINE retrotransposition
Source: PLoS Genet. 2017 Jun 6;13(6):e1006837. doi: 10.1371/journal.pgen.1006837 (PMC5478143; doi:10.1371/journal.pgen.1006837)

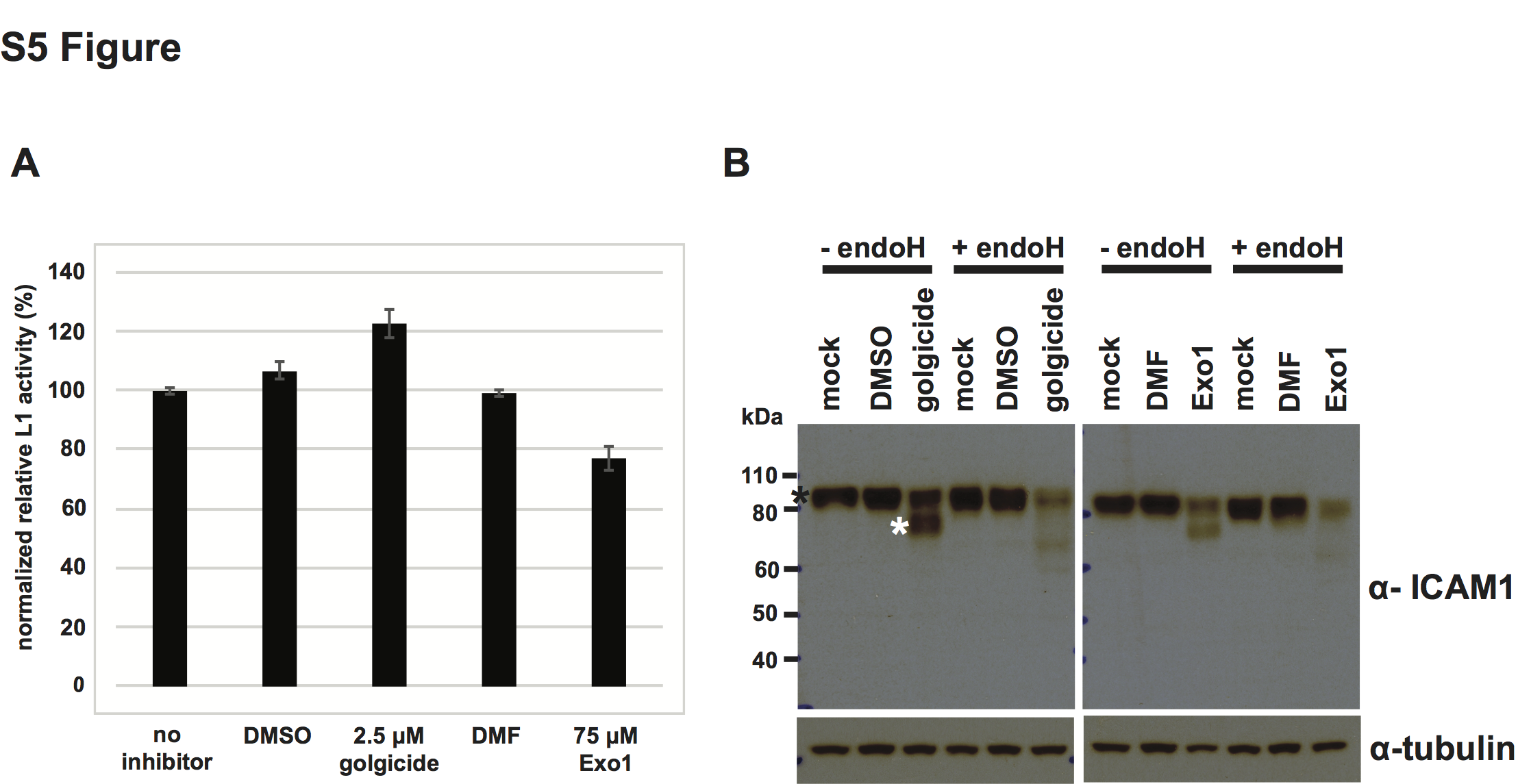

Supplement: S5 Fig — A. Results of L1 retrotransposition assays. HeLa cells were transfected with pJM101L1.3. 1 hour after transfection, media was replaced with media containing the indicated inhibitors. 24 hours after transfection, cells were selected for G418 resistance. DMSO = dimethylsulfoxide. DMF = dimethylformamide. Data normalized for drug toxicity as shown in S8 Table. B. Confirmation of golgi inhibition. HeLa cells were treated with the indicated inhibitors for 24 hours. The cells were lysed and digested with endoglycosidase H. Shown are western blots of undigested (left panel) and digested (right panel) lysates. Inhibition of golgi formation is expected to reduce mature glycosylation of ICAM-1. Black asterisk = mature glycosylated ICAM-1 form. White asterisk = endoH sensitive ICAM-1 form. (TIFF) [file pgen.1006837.s005.tiff]

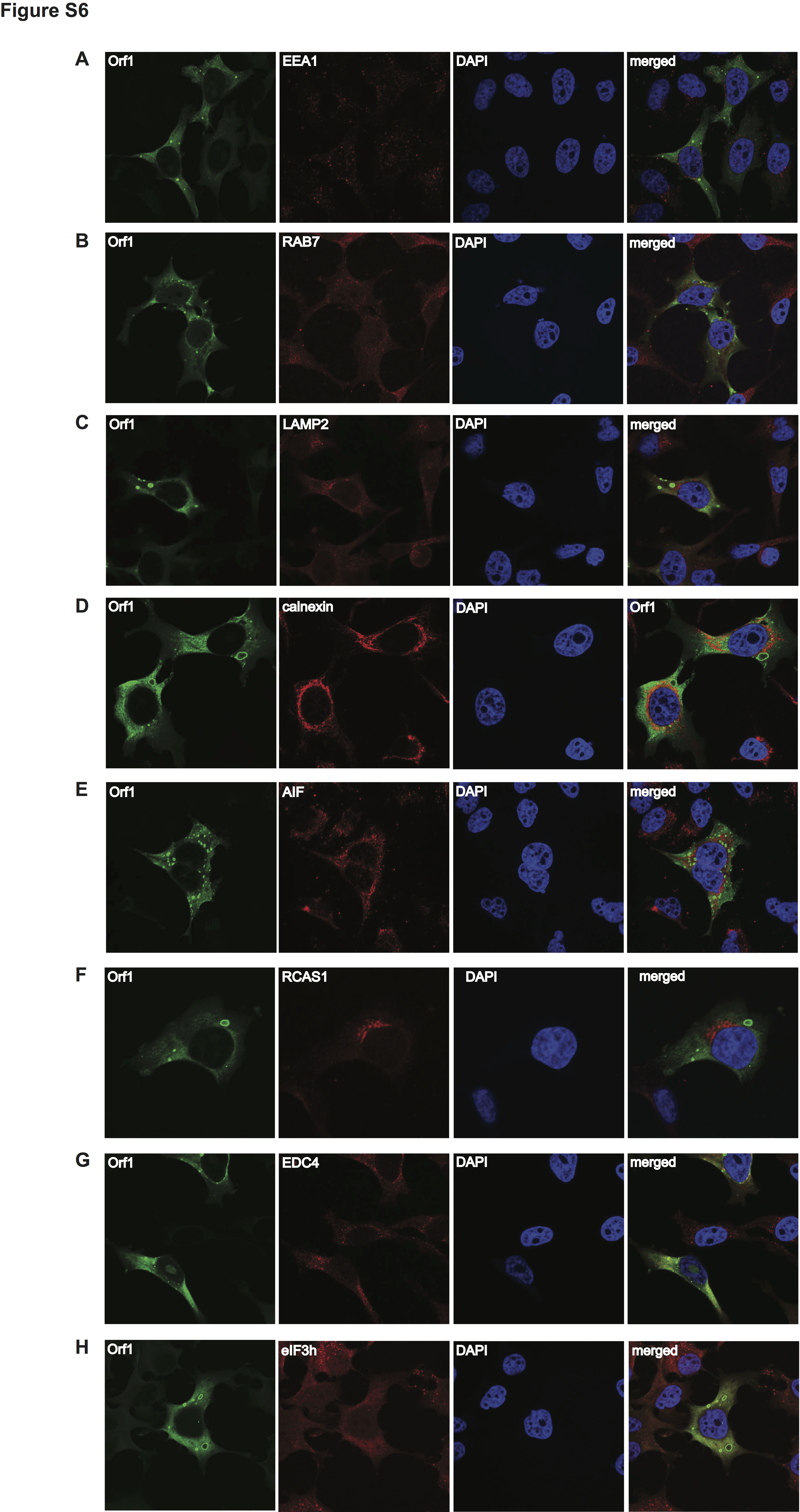

Supplement: S6 Fig — Tet-HeLa cells selected for pTetL1.3 plasmid were treated with 0.5 μg/mL doxycycline for 48 hours to induce L1 expression. At 48 hours, cells were fixed and stained with antibodies against L1 ORF1p and the indicated cellular markers. Alexa Fluor 488 secondary antibodies were used to detect anti-ORF1 antibodies, and Alexa Fluor 594 secondary antibodies were used to detect the cellular markers. DAPI staining shows the nucleus. Shown are representative single planes of confocal imaging. The anti-ORF1p antibody used (JH74 or 4H1) for each subfigure depends on the species source of the cell marker antibody. A. anti-ORF1p and anti-EEA1 (marker for early endosomes). B. anti-ORF1p and anti-RAB7 (marker for late endosomes). C. anti-ORF1 and anti-LAMP2 (marker for lysosomes). D. anti-ORF1 and anti-calnexin (marker for endoplasmic reticulum). E. anti-ORF1 and anti-AIF (marker for mitochondira). F. anti-ORF1 and anti-RCAS1 (marker for golgi). G. anti-ORF1 and anti-EDC4 (marker for P bodies). H. anti-ORF1 and anti-eIF3H (marker for stress granules). (TIFF) [file pgen.1006837.s006.tiff]

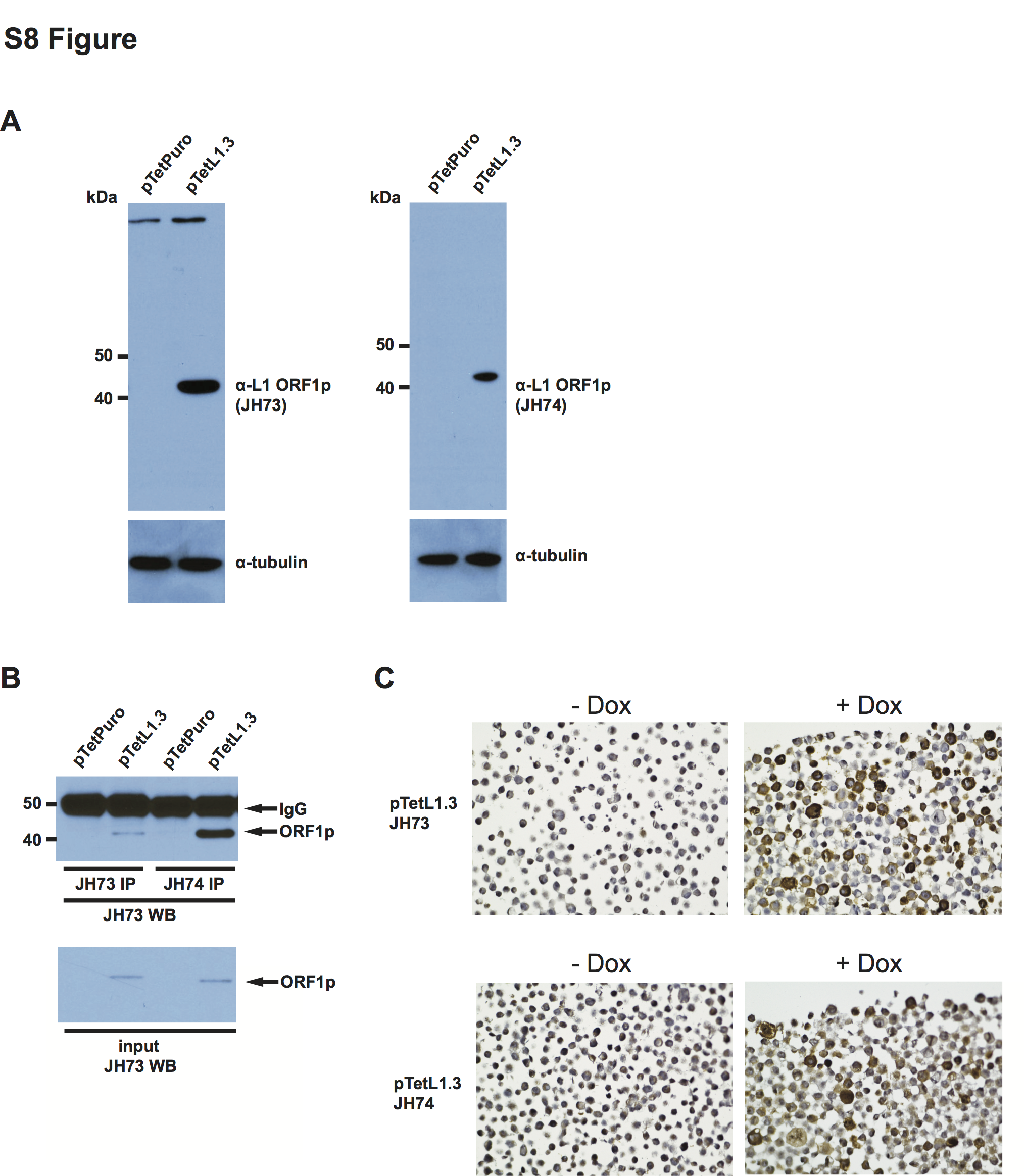

Supplement: S8 Fig — A. Western blots. Tet-HeLa cells were transfected with pTetPuro or pTetL1.3 and selected with puromycin. Puromycin selected pools were treated with doxycycline for 48 hours. Shown are western blots of whole cell lysates harvested at 48 hours. B. Immunoprepcipitations. Tet-HeLa cells were transfected with pTetPuro or pTetL1.3 and selected with puromycin. Puromycin selected pools were treated with doxycycline for 48 hours. Cell lysates taken at 48 hours were immunoprecipitated with JH73 or JH74, then western blotted with JH73. C. Immunohistochemistry. Tet-HeLa cells transfected with pTetL1.3 were treated with or without doxycycline. 48 hours after treatment, cells were fixed with 4% paraformaldehyde, paraffin embedded, and subjected to immunohistochemistry with Vectastain ABD HRP kit and 3,3’-diaminobenzadine tetrahydrochloride (Vector Laboratories). (TIFF) [file pgen.1006837.s008.tiff]
